# Low sensitivity of qSOFA, SIRS criteria and sepsis definition to identify infected patients at risk of complication in the prehospital setting and at the emergency department triage

**DOI:** 10.1186/s13049-017-0449-y

**Published:** 2017-11-03

**Authors:** Selin Tusgul, Pierre-Nicolas Carron, Bertrand Yersin, Thierry Calandra, Fabrice Dami

**Affiliations:** 1 Service of Internal Medicine, Department of Medicine, Lausanne University Hospital, University of Lausanne, Rue du Bugnon 46, -1011 Lausanne, CH Switzerland; 2 Emergency Department, Lausanne University Hospital, University of Lausanne, Rue du Bugnon 46, -1011 Lausanne, CH Switzerland; 3 Infectious Diseases Service, Department of Medicine, Lausanne University Hospital, University of Lausanne, Rue du Bugnon 46, -1011 Lausanne, CH Switzerland

**Keywords:** Sepsis, Septic shock, Quick SOFA score, qSOFA, Prehospital care, Emergency department

## Abstract

**Background:**

Sepsis is defined as life-threatening organ dysfunction caused by a host response to infection. The quick SOFA (qSOFA) score has been recently proposed as a new bedside clinical score to identify patients with suspected infection at risk of complication (intensive care unit (ICU) admission, in-hospital mortality). The aim of this study was to measure the sensitivity of the qSOFA score, SIRS criteria and sepsis definitions to identify the most serious sepsis cases in the prehospital setting and at the emergency department (ED) triage.

**Methods:**

We performed a retrospective study of all patients transported by emergency medical services (EMS) to the Lausanne University Hospital (CHUV) over twelve months. All patients with a suspected or proven infection after the ED workup were included. We retrospectively analysed the sensitivity of the qSOFA score (≥2 criteria), SIRS criteria (≥2 clinical criteria) and sepsis definition (SIRS criteria + one sign of organ dysfunction or hypoperfusion) in the pre-hospital setting and at the ED triage as predictors of ICU admission, ICU stay of ≥3 days and early (i.e. 48 h) mortality. No direct comparison between the three tools was attempted.

**Results:**

Among 11,411 patients transported to the University hospital, 886 (7.8%) were included. In the pre-hospital setting, the sensitivity of qSOFA reached 36.3% for ICU admission, 17.4% for ICU stay of three days or more and 68.0% for 48 h mortality. The sensitivity of SIRS criteria reached 68.8% for ICU admission, 74.6% for ICU stay of three days or more and 64.0% for 48 h mortality. The sensitivity of sepsis definition did not reach 60% for any outcome. At ED triage, the sensitivity of qSOFA reached 31.2% for ICU admission, 30.5% for ICU stay of ≥3 days and 60.0% for mortality at 48 h. The sensitivity of SIRS criteria reached 58.8% for ICU admission, 57.6% for ICU stay of ≥3 days 80.0% for mortality at 48 h. The sensitivity of sepsis definition reached 60.0% for 48 h mortality.

**Discussion:**

Incidence of sepsis in the ED among patients transported by ambulance was 3.8 percent. This rate, associated to the mortality of sepsis, confirms the necessity to dispose of a test to early identify those patients.

**Conclusion:**

The sensitivity performance of all three tools was suboptimal. The qSOFA score, SIRS criteria and sepsis definition have low identification sensitivity in selecting septic patients in the pre-hospital setting or upon arrival in the ED at risk of complication.

## Background

Sepsis is a frequently encountered life-threatening condition with a mortality rate greater than that of acute coronary syndrome or ischemic vascular stroke [1]. In 1992 [2] and 2001 [3], clinical criteria were proposed to define four different stages of sepsis: systemic inflammatory response syndrome (SIRS), sepsis, severe sepsis and septic shock. These criteria were used in the Early Goal-Directed Therapy (EGDT) strategy [4–8]: early recognition of sepsis, improvements in diagnostic procedures and prompt antibiotic therapy were largely implemented, leading to a decrease in overall mortality [9–11].

In 2016, the Sepsis Definition Task Force updated these criteria to increase the specificity for predicting mortality or intensive care unit (ICU) admission. This was done by adapting the criteria to the concepts of pathophysiology (in particular, organ dysfunction), and by removing the concept of SIRS [12]. Regardless of definition, sepsis is still underdiagnosed or diagnosed with delay in the pre-hospital emergency setting and in the emergency department (ED), thereby prejudicing best delivery of care [13–16]. A study of paramedics’ decision-making, for example, considered 30–50% of septic patients as non-urgent [1, 13]. Unfortunately, the existing scores are limited in terms of performance at initial evaluation of patients; the diagnosis of sepsis is therefore still largely dependent on clinical training and physician experience. The SOFA score is intended to be used in the ICU and to a lesser extent in the ED, and is a valuable predictor of severe outcome [17]. Nevertheless, it requires laboratory values, which are usually unavailable in the pre-hospital setting and at ED triage. Owing to these limitations, the task force suggested the use of the “quick SOFA” (qSOFA) score outside of critical care settings, to identify patients with suspected infection who are likely to develop complications of sepsis (ICU admission, in-hospital mortality) [12]. The qSOFA score appears to be a better predictor of in-hospital mortality and ICU admissions for infected patients in the ED than the SOFA score [18]. A recent study also suggested that qSOFA is better than previous criteria at predicting in-hospital mortality among patients with suspected infection in the ED, but in this study, data on the three components of qSOFA were collected at their worst level during the ED stay [19]. Thus, the qSOFA score may be used as an additional tool to prompt prehospital and ED clinicians to consider the possibility of sepsis and escalate care appropriately. For paramedics as well as for ED triage, it is of prime interest to identify a clinical score to recognize the most serious cases among infected patients as early as possible.

The primary aim of this retrospective study was, in the prehospital emergency setting and at ED initial triage, to compare the identification sensitivity of the qSOFA with other clinical criteria or definition (SIRS, sepsis), in selecting, among infected patients, the most severe cases or those at risk of complication (in-hospital mortality at 48 h, admission to the ICU and ICU stay ≥3 days). The secondary goal was to measure the incidence and mortality of septic patients transported by emergency medical services (EMS) to a Swiss urban University Hospital ED.

## Methods

### Study design and setting

We performed a retrospective study on all patients transported by EMS to the Lausanne University Hospital (CHUV), between 1st January and 31st December 2012. The state of Vaud, an area of 3200 km^2^ in western Switzerland, supports a mainly urban and suburban population (730,000 in 2012). A centralised criteria-based dispatch centre covers the whole state and handles c.27,000 primary ambulance transports per year (secondary transfers between hospitals were excluded). There are seven regional hospitals equally distributed throughout the state and one University Hospital, CHUV, a 1050-bed tertiary referral centre. Ambulances are all staffed with emergency medical technicians (EMT) and/or paramedics using state protocols for autonomous intravenous access, cardiopulmonary resuscitation procedures, defibrillation and emergency drug administration. They assess the severity of a patient’s condition according to the National Advisory Committee for Aeronautics (NACA) score [20, 21]. In 2012, the ED triage station of CHUV received 56,836 patients (20% arriving by ambulance); they were all triaged by a four-level emergency scale, the *Swiss Emergency Triage Scale* [22]. The ED admits adults (except ophthalmology and gynaecology patients) and the most critical paediatric cases; there is another specific paediatric ED for less severe cases. Of the 56,836 patients, 36,129 (63.5%) were treated in to the ED, while others were reoriented towards specialist consultants or to the out-patient primary care clinic. The most severe patients were admitted to ICU or ‘intermediate care’ units following their ED work up.

### Population and data sources

The state health services gave us access to the prehospital charts of all patients transported by EMS to the CHUV ED during the year 2012. Patients <18 years old, prisoners, pregnant women, patients in cardio-respiratory arrest, severe trauma victims, and epileptic seizure cases were excluded. The charts, ED and hospital medical records were evaluated by one reviewer. All patients with a suspected infection without alternative diagnosis, or microbiologically proven infection found in the ED workup, were included. A suspicion or diagnosis of infection was made using the following data extracted from prehospital and electronic hospital records in the pre-hospital setting, at ED triage, and then during the first 6 h in the ED: clinical characteristics (systolic blood pressure [SBP], respiratory rate [RR], heart rate [HR], oxygen saturation, body temperature, neurological evaluation (glasgow coma scale (GCS)). Diagnosis on the discharge letter (source of infection, underlying conditions, immune status, outcomes), biological characteristics (laboratory values, microbiological data) and therapy (antibiotic treatment) were also used to include patients. Demographic characteristics (sex, age, date of death) were collected as well. The severity of the infection was assessed within the first 6 h in the ED. This allowed classification of the studied patients into two groups, according to the new definitions of the Third International Consensus Definitions [12]: an infection group (infection without organ dysfunction) and a sepsis group (infection with organ dysfunction, including septic shock). Patients with advance directives requesting limited care were not excluded.

We calculated the qSOFA score and recorded if SIRS and sepsis definitions could be applied to each patient in both groups, at two specific time points: in the prehospital setting with data collected by EMS and at ED triage, with data collected by nurses.

The qSOFA score ranges from 0 to 3 with one point awarded for each of the following clinical signs: SBP ≤100 mmHg, RR ≥22/min, and altered mental status from baseline. A score ≥ 2 signals a greater risk of prolonged ICU stay or increased mortality.

The SIRS criteria use the clinical criteria of the Surviving Sepsis Campaign (SSC) for SIRS [4], comprising at least two of the following criteria: HR >90/min, RR >20/min and temperature < 36° or ≥38.3 °C.

The Sepsis definition used in this work follows the clinical criteria of SSC for severe sepsis [4]: the SIRS definition plus one sign of organ dysfunction or hypoperfusion (GCS < 15 or decline from baseline, oxygen saturation < 90% or SBP <90 mmHg). We analysed the sensitivity of these entities at two specific time points, in the prehospital setting and at the ED’s triage, to predict ICU admission, ICU stay of ≥3 days and mortality at 48 h.

No direct comparison between the three tools was attempted.

### Statistical analysis

Simple descriptive statistics were used to analyze population characteristics. We described data using percentages or medians with interquartile range (IQR). Sensitivities, medians, averages and percentages were calculated using XLSTAT, statistical software compatible with Microsoft Excel (https://www.xlstat.com).

## Results

### Study population

**Fig. 1:**
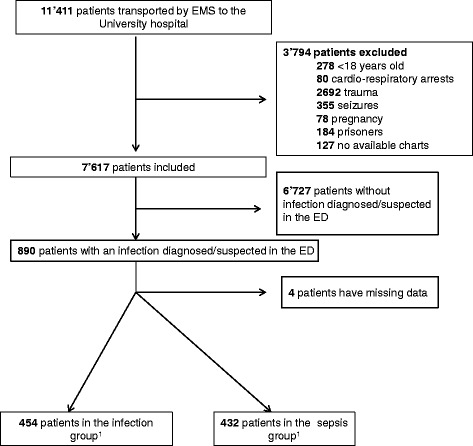
Flowchart. ^1^ Within 6 h in the Emergency Department. Definitions: Infection group: infected patients without organ dysfunction. Sepsis group: including septic shocks; infected patients with organ dysfunction

There were 26,632 primary EMS transports within the State of Vaud in 2012. In total, 11,411 patients were transported to CHUV. In total, 890 patients fulfilled the criteria of a diagnosis or suspicion of infection in the ED; four had missing data and were excluded. Finally, 886 (7.8%) patients were included (Fig. 1).

### General characteristics

In all, 462 (52.1%) patients were male, the mean age was 80 years (range: 22–102), and 131 (14.8%) were immunosuppressed (Table 1). Following the ED workup, 454 (51.2%) patients were classified in the infection group and 432 (48.8%) in the sepsis group (Fig. 1). The incidence of sepsis in the ED among patients transported by ambulance to CHUV was 3.8/100 patients.

**Table 1:** Patient characteristics

		Severity of the infection within 6 h of ED admission	
	All patients	Infection group	Sepsis group
Demographics			
N (%)	886 (100)	454 (51.2)	432 (48.8)
Male (%)	462 (52.1)	232 (51.1)	230 (53.2)
Mean age (min-max)	80 (22–102)	80 (22–100)	79 (25–102)
Immunosuppression^a^ (%)	131 (14.8)	53 (11.7)	78 (18.1)
Outcomes			
ICU admissions (%)	80 (9.0)	7 (1.5)	73 (16.9)
ICU stay of ≥3 days (%)	59 (73.8)	4 (57.1)	55 (75.3)
Mortality at 48 h (%)	25 (2.8)	2 (0.4)	23 (5.3)
Focus of infection			
Respiratory (%)	486 (54.9)	209 (46.0)	277 (64.1)
Genitourinatory (%)	178 (20.1)	111 (24.5)	67 (15.5)
Gastrointestinal (%)	115 (13.0)	74 (16.3)	41 (9.5)
Skin/joint (%)	66 (7.5)	48 (10.6)	18 (4.2)
Central nervous system (%)	8 (0.9)	1 (0.2)	7 (1.6)
Endocarditis (%)	4 (0.5)	2 (0.4)	2 (0.5)
Other (%)	17 (1.9)	5 (1.1)	12 (2.8)
Unknown^b^ (%)	12 (1.4)	5 (1.1)	7 (1.6)

Data are presented as n (%)

Infection group: infected patients without organ dysfunction. Sepsis group: including septic shocks; infected patients with organ dysfunction

*ED* emergency department, *ICU* intensive care unit

^a^Included human immunodeficiency virus, chronic steroid use (>1 month) and cancer

^b^Infection suspected, but no pathogen and/or site identified

Eighty patients (9.0%) were admitted to the ICU (16.9% of the sepsis group (73) and 1.5% of the infection group (7)), of whom 59 (73.8%) stayed in the ICU for ≥3 days (75.3% of the sepsis group (55) and 57.1% of the infection group (4)) (Table 1). Twenty-five (2.8%) patients died within 48 h after admission (5.3% of the sepsis group (23) and 0.4% of the infection group (2)) (Table 1). The site of infection was respiratory in 486 patients (54.9%), genitourinary in 178 (20.0%), gastrointestinal in 115 (13.0%) and skin and joint in 66 (7.4%) (Table 1). Infections were microbiologically documented in 354 patients (40%). The most commonly encountered pathogens were *Escherichia coli* (*n* = 137, 38.7%), *Streptococci* (*n* = 61, 17.2%), and *Staphylococcus aureus* (*n* = 27, 7.6%). Polymicrobial flora was found in 40 cases (11.3%).

Table 2 shows the numbers and rates of patients corresponding to the clinical entities studied correlated to the outcomes.

**Table 2:** Clinical tools

	All patients	ICU admission	ICU stay ≥ 3 days	Mortality at 48 h
N (%)	886 (100)	80 (9)	59 (73.8)	25 (2.8)
Prehospital setting				
qSOFA score ≥ 2 (%)	165 (18.6)	29 (36.3)	8 (13.6)	17 (68.0)
SIRS criteria (%)	437 (49.3)	55 (68.8)	44 (74.6)	16 (64.0)
Sepsis definition (%)	234 (26.4)	40 (50.0)	32 (54.2)	13 (52.0)
ED’s triage				
qSOFA score ≥ 2 (%)	149 (16.8)	25 (31.3)	18 (30.5)	15 (60.0)
SIRS criteria (%)	374 (42.2)	47 (58.8)	34 (57.6)	20 (80.0)
Sepsis definition (%)	221 (24.9)	34 (42.5)	25 (42.4)	15 (60.0)

Data are presented as n (%)

qSOFA score: ≥2 items: RR ≥22/min; SBP ≤100 mmHg; GCS <15 or decline from baseline. SIRS criteria: ≥2 items: RR >20/min, HR >90/min, temperature ≥ 38.3 °C or <36 °C. Sepsis definition: SIRS criteria plus one of the following item: SBP <90 mmHG, GCS < 15 or decline from baseline, oxygen saturation < 90%

*ED* emergency department, *ICU* intensive care unit

### Sensitivity of the clinical tools in the prehospital setting

The sensitivity of qSOFA (≥2) reached 36.3% for ICU admission, 17.4% for ICU stay of ≥3 days and 68% for 48 h mortality. The sensitivity of SIRS criteria reached 68.8% for ICU admission, 74.6% for ICU stay of ≥3 days and 64% for 48 h mortality. The sensitivity of sepsis definition did not reach 60% for any outcome (Table 3).

**Table 3:** Sensitivity of clinical tools to predict clinical outcomes

	ICU admission	ICU stay ≥ 3 days	Mortality at 48 h
Prehospital setting (%;IQR)			
qSOFA score ≥ 2	36.3 (26.6–47.2)	17.4 (9.1–30.7)	68.0 (48.4–82.8)
SIRS criteria	68.8 (57.9–77.9)	74.6 (62.2–84)	64.0 (44.5–79.8)
Sepsis definition	50 (39.3–60.7)	54.2 (41.7–66.3)	52.0 (33.5–70)
ED’s triage (%;IQR)			
qSOFA score ≥ 2	31.2 (22.2–42.1)	30.5 (20.3–43.2)	60.0 (40.7–76.6)
SIRS criteria	58.8 (47.8–68.9)	57.6 (44.9–69.4)	80.0 (60.9–91.1)
Sepsis definition	42.5 (32.3–53.4)	42.4 (30.6–55.1%)	60.0 (40.7–76.6)

Data are presented as percentages and interquartile range (IQR)

qSOFA score: ≥2 items: RR ≥22/min; SBP ≤100 mmHg; GCS <15 or decline from baseline. SIRS criteria: ≥2 items: RR >20/min, HR >90/min, temperature ≥ 38.3 °C or <36 °C. Sepsis definition: SIRS criteria plus one of the following item: SBP <90 mmHG, GCS <15 or decline from baseline, oxygen saturation < 90%

*ED* emergency department, *ICU* intensive care unit

### Sensitivity of the clinical tools at ED triage

The sensitivity of qSOFA (≥2) reached 31.2% for ICU admission, 30.5% for ICU stay of ≥3 dayse and 60% for 48 h mortality. The sensitivity of SIRS criteria reached 58.8% or ICU admission, 57.6% for ICU stay of ≥3 days and 80.0% for mortality at 48 h. The sensitivity of sepsis definition reached 60.0% for 48 h mortality. All other measured values did not reach 60% for any outcome (Table 3).

## Discussion

Incidence of sepsis in the ED among patients transported by ambulance to CHUV is 3.8 per 100 patients. The high number of patients admitted with sepsis confirms the importance of this diagnosis in terms of volume and severity. Thus early recognition, which allows the possibility of initiating early treatment, is important [1, 11].

The qSOFA is a relatively new clinical score used to identify septic patients outside the critical care unit likely to develop complications. It has been validated in a prospective multicentric study for patients already ongoing ED work-up [19].

For patients in the pre-hospital setting or in the ED with infection diagnosed or suspected afterward, we retrospectively calculated the sensitivity of the qSOFA score, SIRS criteria and sepsis definitions to identify cases at risk of complication. In summary, the sensitivity performance of all three tools was suboptimal. The qSOFA score, in particular, does not achieve in recognizing the most seriously infected patients early, and so brings no extra value to paramedics or prehospital physicians in their quest to identify such. A similar conclusion was reached by Dorsett et al. [23], but on a much smaller population than in our study.

At ED triage, as previously reported [24, 25], qSOFA scored poorly in identifying severe sepsis, and was likewise poor in both pre-hospital and at ED triage in predicting ICU stays ≥3 days. In both settings, it was best in identifying infected patients at risk of mortality at 48 h.

The sepsis definition also performed poorly, as its sensitivity never exceeded 60%. However, the SIRS criteria performed well, both in the prehospital setting and at the ED triage, with sensitivity results from 68 to 80%, probably owing to the absence of specificity for organ dysfunction, which exists already at an advanced stage of infection. The SSC considers that SIRS criteria remain useful for the identification of infection [26]. Williams et al. go even further by establishing that SIRS is associated with an increased risk of deleterious response to infection and mortality, and would be a useful screening tool [27]. In the meantime, for the prehospital setting in particular, we are still awaiting a validated sensitive clinical tool to identify early septic patients of any severity, let alone those at risk.

## Limitations

Ours is a monocentric, retrospective study, conducted in a specific prehospital setting. qSOFA score, SIRS and sepsis definitions were only applied to confirmed or suspected infected patients within the ED; therefore, sensitivity was the sole performance measured. A sole reviewer determined the clinical categories and final diagnosis.

## Conclusion

The qSOFA score, SIRS criteria and sepsis definition exhibited suboptimal performance for early recognition of infected patients at risk of complication in the prehospital setting or at ED triage. There is still a lack of a clinical tool to help prehospital caregivers and ED clinicians to identify early, prior to any laboratory results, infected patients at greater risk of poor outcome.

## Abbreviations

CHUV: Lausanne University Hospital
ED: Emergency department
EGDT: Early goal-directed therapy
EMS: Emergency medical services
EMT: Emergency medical technicians
GCS: Glasgow coma scale
HR: Heart rate
ICU: Intensive care unit
MAP: Mean arterial pressure
qSOFA: quick SOFA score
RR: Respiratory rate
SBP: Systolic blood pressure
SIRS: Systemic inflammatory response syndrome
SOFA: Sepsis-related Organ Failure Assessment
SSC: Surviving sepsis campaign
